# Azathioprine Metabolites in Erythrocytes and DNA for Therapy Monitoring in Very Early Onset Inflammatory Bowel Disease Pediatric Patients

**DOI:** 10.1021/acsptsci.5c00135

**Published:** 2025-06-05

**Authors:** Giulia Zudeh, Martina Franzin, Marianna Lucafò, Matteo Bramuzzo, Debora Curci, Jun J. Yang, Maud Maillard, Giuliana Decorti, Gabriele Stocco

**Affiliations:** † Department of Translational and Advanced Diagnostics, Institute for Maternal and Child Health I.R.C.C.S. Burlo Garofolo, Trieste 34137, Italy; ‡ Department of Life Sciences, 9315 University of Trieste, 34128 Trieste, Italy; § Department of Gastroenterology, Digestive Endoscopy and Nutrition Unit, Institute for Maternal and Child Health I.R.C.C.S. Burlo Garofolo, Trieste 34137, Italy; ∥ Department of Pharmacy and Pharmaceutical Sciences, St. Jude Children’s Research Hospital, Memphis, Tennessee 38105-3678, United States; ⊥ Department of Medical, Surgical and Health Sciences, University of Trieste, Trieste 34129, Italy

**Keywords:** very early onset inflammatory bowel disease, azathioprine, DNA-TG, TGN, pharmacogenetics, pediatric

## Abstract

Azathioprine is used for inflammatory bowel disease (IBD) therapy. Patients under 6 years of age (very early onset, VEO-IBD) showed distinctive clinical characteristics, such as increased activity of thiopurine-methyltransferase (TPMT), a crucial enzyme for thiopurine metabolism. *TPMT* and *PACSIN2* polymorphisms were associated with azathioprine efficacy. This study investigated the role of age in thiopurine active metabolites and disease activity. Also, the effects of age, *TPMT* and *PACSIN2* polymorphisms on azathioprine metabolites and disease activity were evaluated. Erythrocytes thioguanine nucleotides (TGN) were measured by HPLC, and leukocytes incorporated deoxythioguanosine (DNA-TG) byLC-MS/MS in 12 VEO-IBD patients (median age 4.13 ± 0.98, 7 females, 6 Crohn’s disease (CD)), 11 IBD children (median age 9.36 ± 1.52, 8 females, 1 CD ), and 73 IBD adolescents (median age 14.92 ± 1.81, 33 females, 37 CD). VEO-IBD subjects required a higher azathioprine dose (*p*-value = 0.048) and showed a lower DNA-TG/azathioprine dose ratio (*p*-value = 0.049) and TGN/azathioprine dose ratio (*p*-value = 0.013). DNA-TG was positively correlated with TGN (*p*-value = 4.15 × 10^–5^) and disease score (*p*-value = 1.54 × 10^–4^). *TPMT* rs1142345 (76 wild type, 10 heterozygous) was associated with increased concentrations of DNA-TG and TGN (*p*-value = 0.024 and 0.00038, respectively), whereas *PACSIN2* rs2413739 (37 wild types, 34 heterozygous, 16 homozygous variants) was associated with the disease score (*p*-value = 0.04). Together, these data confirmed that VEO-IBD patients show enhanced azathioprine metabolism, which can be accurately reflected by both TGN and DNA-TG levels, highlighting their ability to be good biomarkers of azathioprine metabolism.

## Graphical abstract

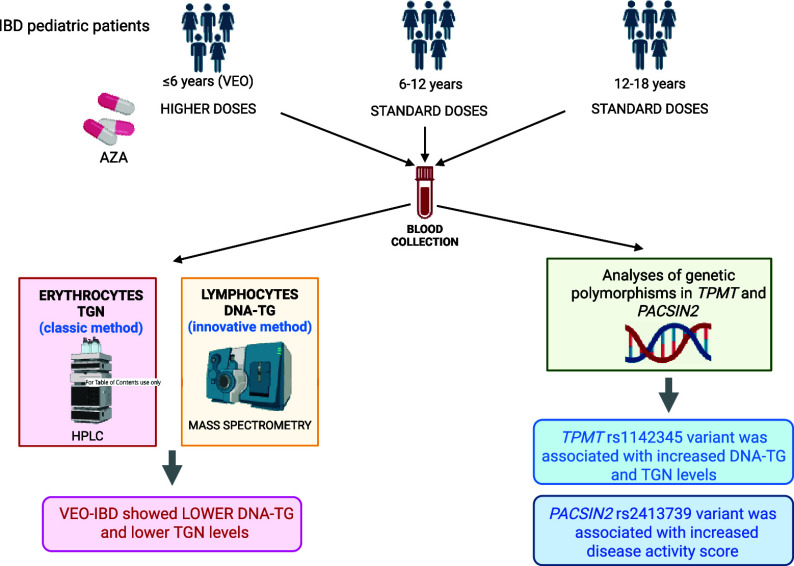

Inflammatory bowel diseases (IBD) are a group of disorders involving inflammation of the gastrointestinal tract, mainly represented by Crohn’s disease (CD) and ulcerative colitis (UC). Despite IBD presenting with different onset ages, around 25% of cases occur during childhood and adolescence.^1^ Even more cases show a “very early onset” (VEO-IBD) and are diagnosed before the sixth year of life, representing 5–15% of IBD patients.^2^ VEO-IBD patients present some peculiar characteristics, such as a predominant colonic involvement, a high genetic predisposition, and a more aggressive disease behavior with an increased risk of adverse effects on growth.^2,3^ In recent years, even more evidence has demonstrated that VEO-IBD patients also present some peculiarity in the pharmacokinetics of the administered drugs, such as azathioprine, one of the most used immunosuppressants utilized to maintain disease remission.^4^ Azathioprine is metabolized through a complex pathway, in which the enzyme thiopurine-methyltransferase (TPMT) plays a crucial role in azathioprine inactivation. TPMT presents genetically determined interindividual variability, and its variants are associated with the development of azathioprine side effects in IBD pediatric patients.^5^ Previous studies have also associated the *PACSIN2* rs2413739 variant with azathioprine efficacy.^6^ Interestingly, it has been found that age affects TPMT enzyme activity, which is increased in VEO-IBD subjects, leading to the requirement of higher azathioprine doses to reach the correct thioguanine nucleotide (TGN) therapeutic range.^7^ Thiopurines exert their cytotoxicity after being extensively metabolized into TGN, which are further incorporated into nucleic acids and trigger apoptosis. Despite lymphocytes being the main thiopurine target, TGN is usually measured in red blood cells (RBC) using different protocols based on high-performance liquid chromatographic (HPLC) assays.^7,8^

Thanks to the recent improvement in liquid chromatography tandem mass spectrometry (LC-MS/MS) techniques, the evaluation of incorporated deoxythioguanosine (DNA-TG) levels in white blood cells (WBC) may provide a more accurate indication of drug efficacy and could be considered a promising marker to evaluate both treatment success and the risk of toxicity development.^9,10^ In this study on IBD pediatric patients, the association between TGN levels in RBC and DNA-TG levels in WBC was performed, and the correlations between their concentrations and the disease activity score were investigated; also, the effect of patient age on the administered azathioprine doses was tested. Finally, the possible impact of genetic polymorphisms in *TPMT* and *PACSIN2* on TGN and DNA-TG levels was evaluated.

## Results

### Patients

Ninety-six samples from 70 IBD pediatric patients were collected: 12 samples derived from 10 VEO-IBD patients, 11 samples derived from 8 children between 6 and 12 years, and 73 samples derived from 52 adolescents. Demographic and clinical characteristics are reported in Table 1; in particular, the reported data about sex and disease type referred to the 70 enrolled patients, whereas both age and disease activity scores at the moment of the blood sample collection referred to all 96 samples included in the study.

**Table 1:** Demographic and Clinical Characteristics^a^

demographic and clinical parameters		
sample number	under 6 (VEO-IBD)	12
	6–12 (children)	11
	12–18 (adolescents)	73
	total	96
sex	under 6 (VEO-IBD)	5 F – 5 M
	6–12 (children)	5 F – 3 M
	12–18 (adolescents)	26 F – 26 M
	total	36 F – 34 M
IBD type	under 6 (VEO-IBD)	4 CD – 6 UC
	6–12 (children)	1 CD – 7 UC
	12–18 (adolescents)	25 CD – 27 UC
	total	30 CD – 40 UC
age (mean ± SD)	under 6 (VEO-IBD)	4.13 ± 0.98
	6–12 (children)	9.36 ± 1.52
	12–18 (adolescents)	14.92 ± 1.81
	total	12.93 ± 4.14
PCDAI (median, IQR)	under 6 (VEO-IBD)	0, 0
	6–12 (children)	2.5, 0
	12–18 (adolescents)	2.5, 7.5
	total	2.5, 7.5
PUCAI (median, IQR)	under 6 (VEO-IBD)	7.5, 0
	6–12 (children)	0, 0–0
	12–18 (adolescents)	0, 0–1.25
	total	0, 0–4.37

a The reported data about sex and disease type referred to the 70 enrolled patients, whereas both age and disease activity scores at the moment of the blood sample collection referred to all 96 samples included in the study. Abbreviations: Crohn’s disease, CD; inflammatory bowel disease, IBD; interquartile range, IQR; pediatric ulcerative colitis activity index, PUCAI; pediatric Crohn’s disease activity index, PCDAI; ulcerative colitis, UC; very early onset IBD, VEO-IBD.

Data normality was checked using the Shapiro test (Figure S1).

There were no significant associations between IBD type and WBC DNA-TG and RBC TGN concentrations (Wilcoxon *p-*value = 0.66 and Wilcoxon *p-*value= 0.4, Figure S2) or between patients’ gender and WBC DNA-TG and RBC TGN (Wilcoxon *p-*value = 0.06 and Wilcoxon *p*-value = 0.7, Figure S3).

### Effect of Age on Azathioprine Dosage

The administered azathioprine dosage was available for 95 samples after an average of 451 days (IQR 1062.25) of azathioprine therapy. A significant effect of patients’ age on the administered azathioprine dose (mg/kg) was detected: the VEO-IBD subjects required a significantly higher drug dosage (median dose of 2.22 mg/kg, IQR 0.44) compared to patients between 6 and 12 years (median dose of 1.97 mg/kg, IQR 0.53) and IBD adolescents (median dose of 1.88 mg/kg, IQR 0.83) (Kruskal–Wallis *p*-value = 0.048, Figure 1). A similar trend was found when analyses were adjusted for repeated measures in each patient (linear mixed effect model *p*value = 0.088), confirming the role of age in azathioprine pharmacokinetics, which is higher in younger patients.

**Figure 1:**
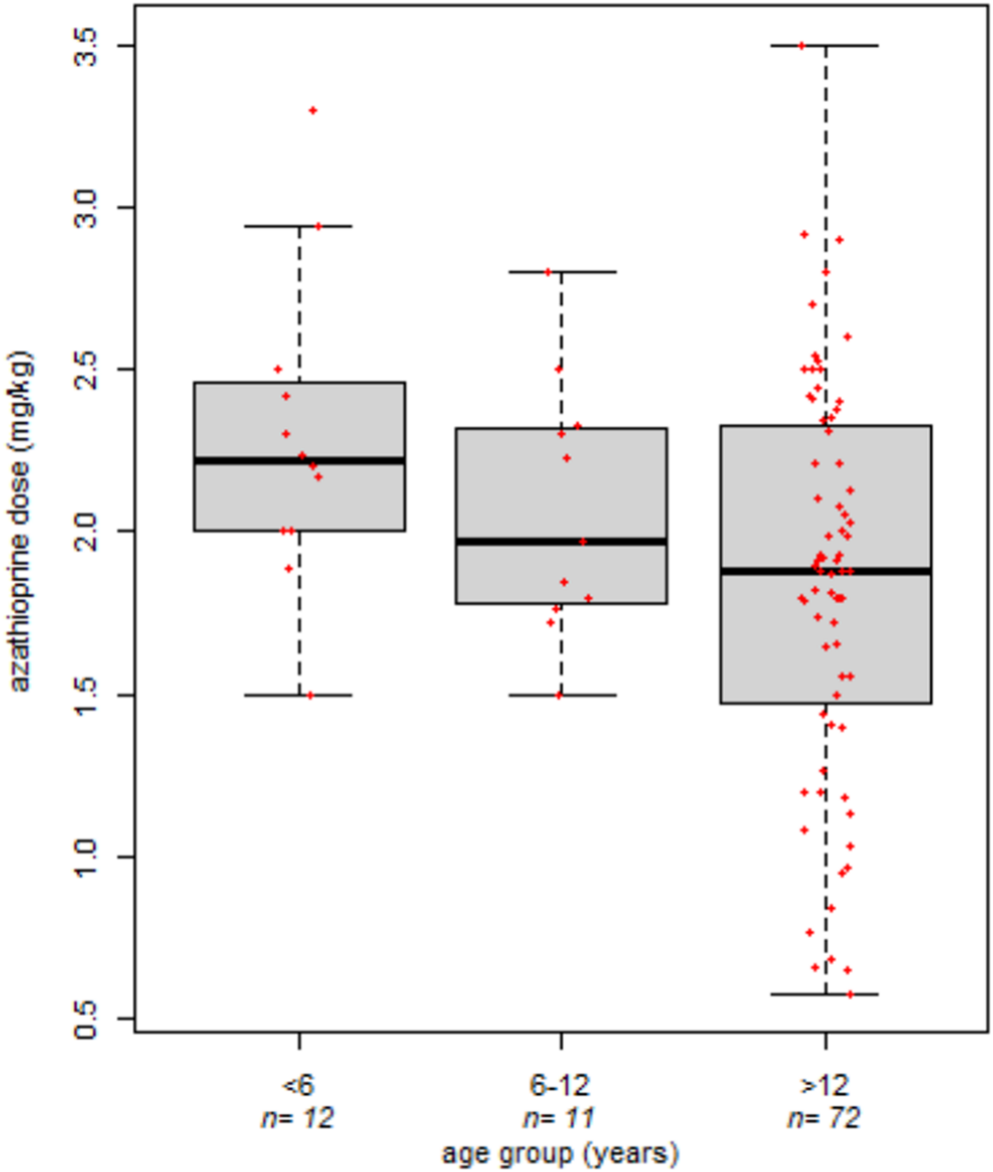
Effect of age on the administered azathioprine dose (mg/kg) in IBD pediatric patients, Kruskal–Wallis *p*-value = 0.048.

### Impact of Age on the DNA-TG Level

A trend demonstrating a decreased DNA-TG concentration in VEO-IBD patients (median 224.2 fmol/μgDNA, IQR 232.68 fmol/μgDNA) compared to adolescent IBD patients (median 349.82 fmol/μgDNA, IQR 379.15 fmol/μgDNA) was detected, whereas similar DNA-TG concentrations were found between VEO-IBD patients and subjects between 6 and 12 years (median 223.67 fmol/μgDNA, IQR 164.31 fmol/μgDNA) (Figure 2a).

**Figure 2:**
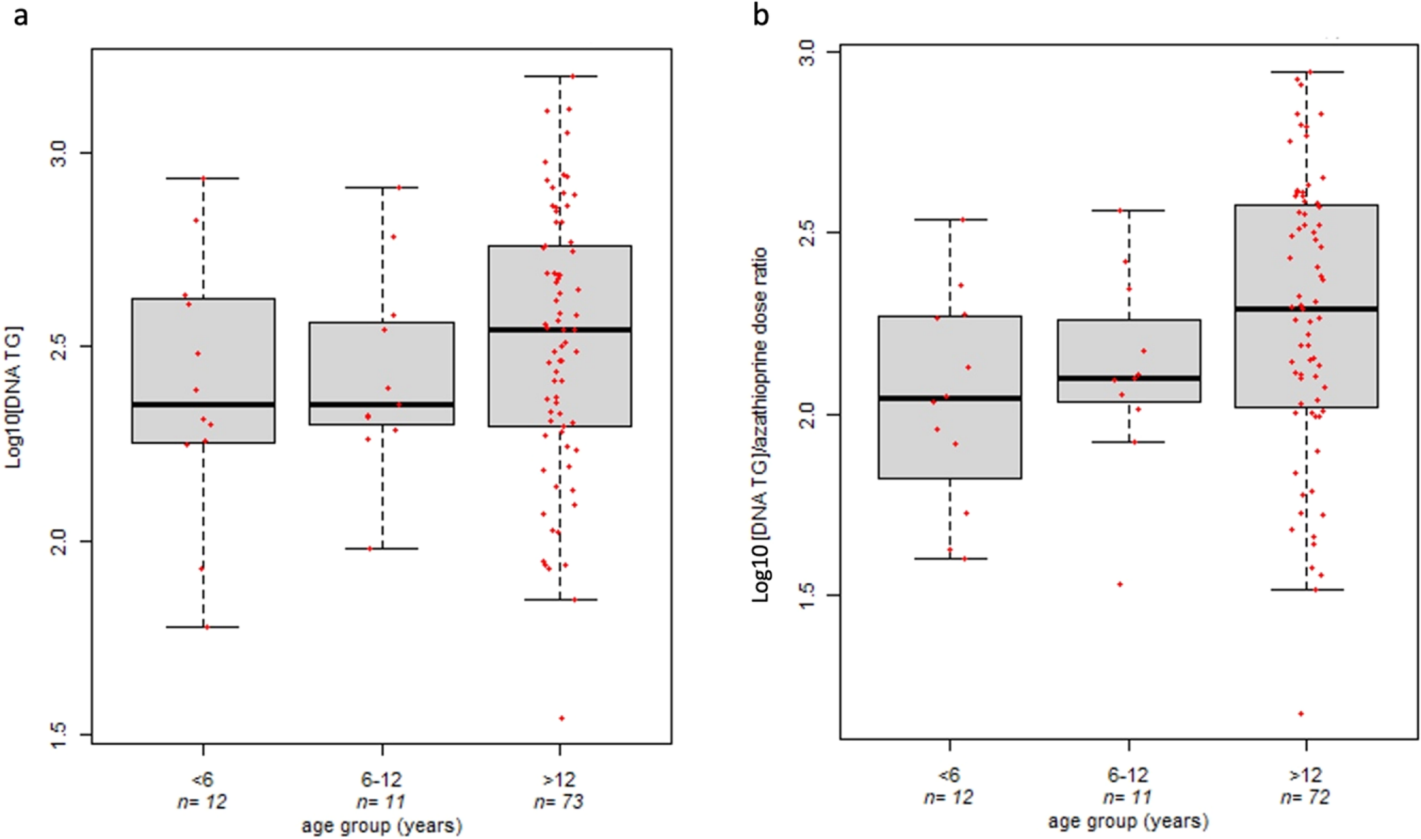
Impact of age on the WBC DNA-TG levels, Kruskal–Wallis *p*-value = 0.36 (panel a), and the ratio between DNA-TG levels and azathioprine dose in IBD pediatric patients, Kruskal–Wallis *p*-value = 0.048 (panel b).

After adjusting the DNA-TG amount for the administered azathioprine dosage, a significant effect of age emerged in the analyzed cohort. In particular, a significant reduction in the ratio between DNA-TG concentration and azathioprine dose was found in VEO-IBD patients (median 110.32 fmol/μgDNA/mg/kg, IQR 110.13 fmol/μgDNA/mg/kg) compared to both IBD patients between 6 and 12 years (median 125.92 fmol/μgDNA/mg/kg, IQR 77.05 fmol/μgDNA/mg/kg) and IBD adolescents (median 196 fmol/μgDNA/mg/kg, IQR 270.53 fmol/μgDNA/mg/kg) (Kruskal–Wallis *p*-value = 0.049, Figure 2b). A similar trend was found when adjusting the analyses for repeated measures in each patient (linear mixed effect model *p*-value = 0.10).

### Effect of Age on RBC TGN Concentration

A trend demonstrating a lower concentration of TGN metabolites in RBC of VEO-IBD patients (median 200.5 pmol/8 × 10^8^ erythrocytes, IQR 150.5 pmol/8 × 10^8^ erythrocytes) compared to both patients between 6 and 12 years old (median 300 pmol/8 × 10^8^ erythrocytes, IQR 156.27 pmol/8 × 10^8^ erythrocytes) and IBD adolescents (median 342.22 pmol/8 × 10^8^ erythrocytes, IQR 280.04 pmol/8 × 10^8^ erythrocytes) was found, showing also an age scalar effect (Figure 3a).

**Figure 3:**
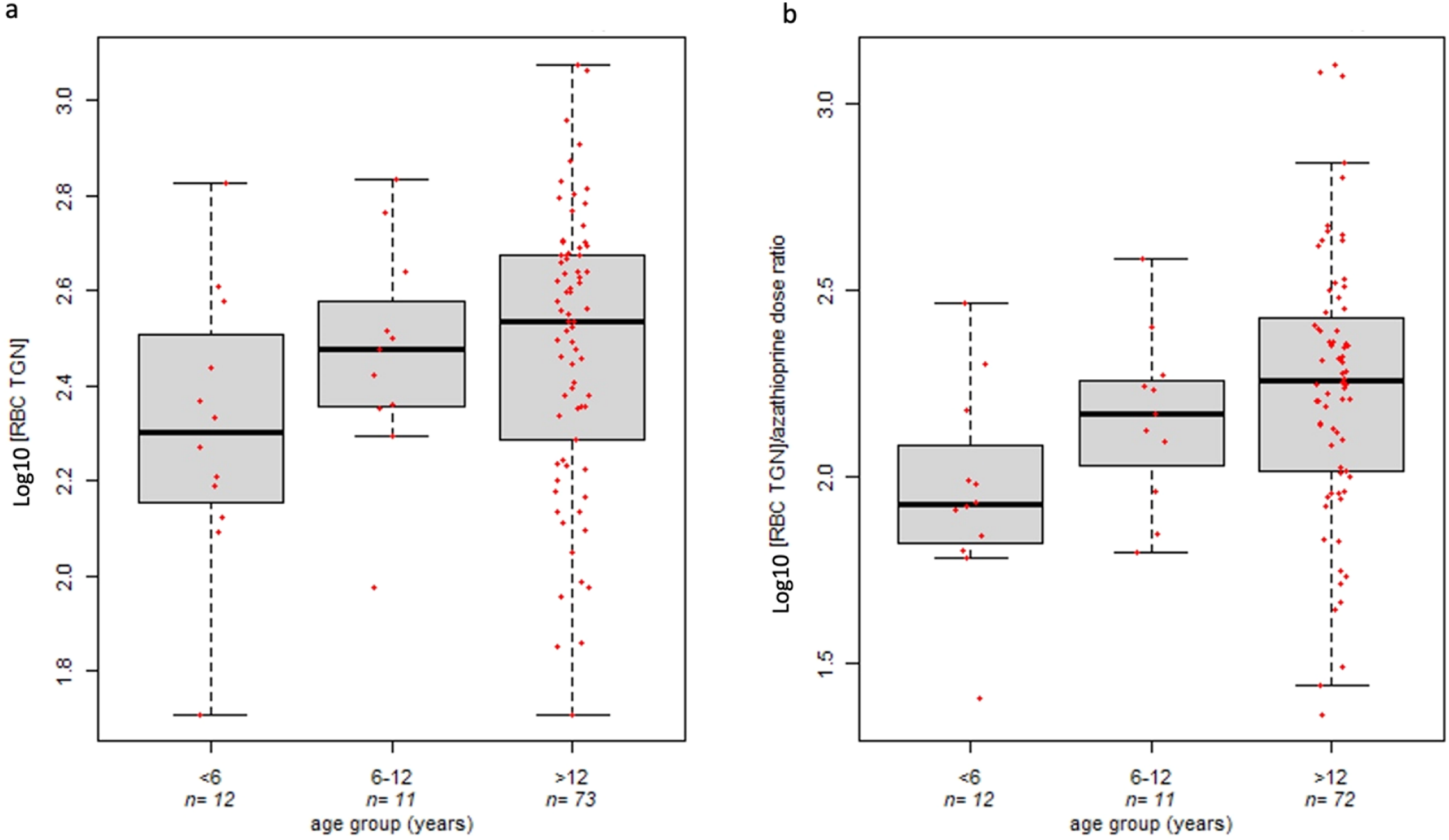
Effect of age on the TGN levels measured in RBC, Kruskal–Wallis *p*-value = 0.12 (panel a), and the ratio between TGN levels and azathioprine dose in IBD pediatric patients, Kruskal–Wallis *p*-value = 0.013 (panel b).

The amount of these azathioprine metabolites adjusted for the administered drug dosage showed a significant effect of age; VEO-IBD patients presented a lower TGN/azathioprine dose ratio (median of 84.11, IQR 43.3) than subjects between 6 and 12 years (median of 147.05, IQR 73.02) and IBD adolescents (median of 180.87, IQR 157.5) (Kruskal–Wallis *p*-value = 0.013). A comparable trend was identified by adjusting the analyses for repeated measures in each patient (linear mixed effect model *p*-value = 0.068).

### Correlation between DNA-TG and TGN Levels

In order to evaluate the possible association between WBC DNA-TG and RBC TGN concentrations, a correlation analysis was performed on the 96 observations of the 70 enrolled patients, and a significant positive correlation was found (ρ = 0.41, Spearman’s *p*-value = 4.15 × 10^–5^, Figure 4). A comparable trend was identified, adjusting the analyses for repeated measures in each patient (linear mixed effect model *p*-value = 3 × 10^–4^)

**Figure 4:**
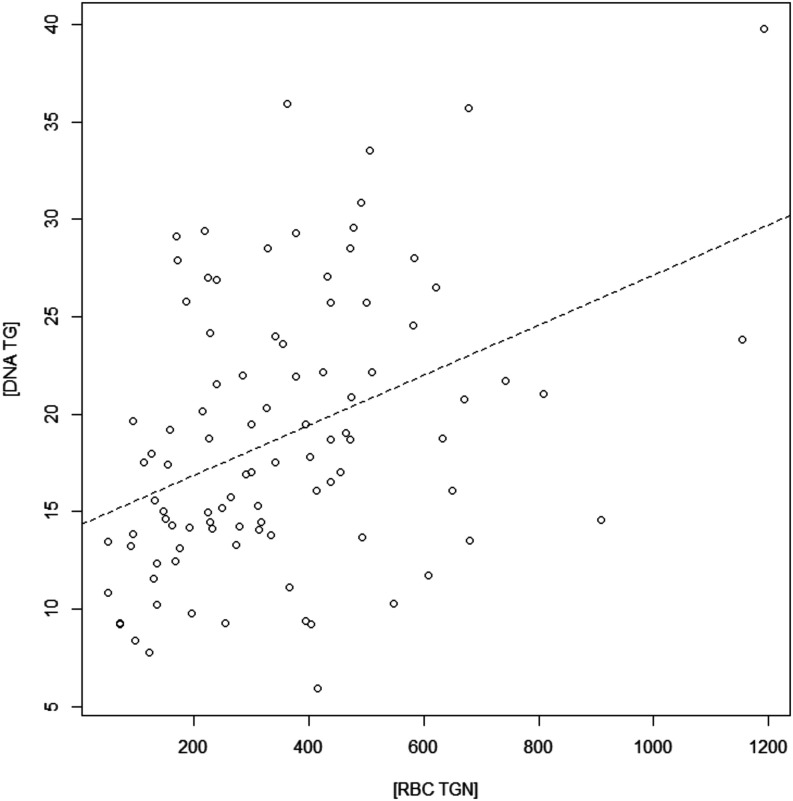
Association between WBC DNA-TG and RBC TGN levels in 96 observations of 70 IBD pediatric patients. Spearman ρ = 0.41 and *p*-value = 4.15 × 10^–5^.

### Correlations between DNA-TG and TGN Levels, Azathioprine Dose, Disease Activity Score, and Clinical Parameters of Azathioprine Toxicity

The administered azathioprine dose did not correlate with the WBC DNA-TG amount (Spearman ρ = −0.024, *p*-value = 0.82, Figure S4a) or RBC TGN concentration (Spearman ρ = −0.086, *p* = 0.41, Figure S4b). WBC DNA-TG levels were found to be positively correlated with the disease activity score (ρ = 0.38, Spearman *p*-value = 1.54 × 10^–4^, Figure 5a), whereas no associations between RBC TGN and the clinical disease scores were detected (Figure 5b). Similar patterns for the association with the disease activity score were observed for WBC DNA-TG/azathioprine dose ratio (Spearman ρ = 0.35, *p*-value= 0.00059, Figure S5a) and RBC TGN/azathioprine dose ratio (Spearman ρ = 0.016, *p-value* = 0.88, Figure S5b), whereas the azathioprine dose was not associated with the disease activity score (Figure S6).

**Figure 5:**
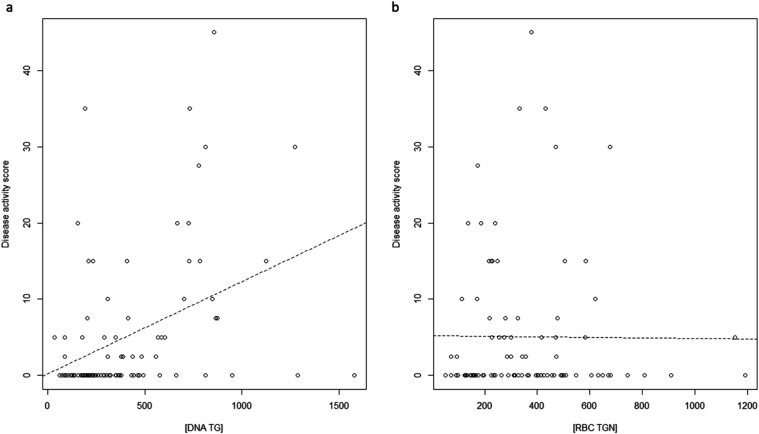
Correlation analysis between DNA-TG levels and the disease activity score, Spearman ρ = 0.38, *p*-value = 1.54 × 10 ^–4^ (panel a); and correlation analysis between TGN levels and the disease activity score (panel b).

The possible correlations between the azathioprine metabolites and the clinical parameters used for determining azathioprine toxicity (leukocyte, erythrocyte, and platelet counts, hemoglobin concentration, mean corpuscular volume (MCV), liver enzymes alanine aminotransferase (ALT), aspartate aminotransferase (AST), and γ-glutamyltransferase (GGT), and amylase levels) were tested. The WBC DNA-TG levels correlated negatively with the lymphocyte count (Spearman ρ = −0.24, *p*-value= 0.019, Figure S7a) and amylase (Spearman ρ = −0.3, *p*-value = 0.026, Figure S7b), whereas it showed a positive association with the level of mean corpuscular volume (MCV, Spearman ρ = 0.24, *p*-value= 0.05, Figure S7c). The TGN amount negatively correlated with the WBC count (Spearman ρ = −0.4, *p*-value = 1.48 × 10^–5^, Figure S8a), neutrophil count (Spearman ρ = −0.3, *p*-value = 0.03, Figure S8b), lymphocyte count (Spearman ρ = −0.2, *p*-value = 0.03, Figure S8c), and platelet count (Spearman ρ = −0.4, *p*-value = 0.0015, Figure S8d), whereas the TGN level was positively correlated with MCV (Spearman ρ = 0.3, *p*-value = 0.02, Figure S8d).

### Role of *TPMT* and *PACSIN2* Genetic Variants on Azathioprine Metabolite Levels and the Disease Activity Scores

*TPMT* genotypes were available for 61 subjects for a total of 86 observations, whereas the *PACSIN2* genotype was tested in 61 patients for a total of 87 observations (Table S1). Regarding *TPMT* alleles, 55 patients were classified as *1/*1, 5 as *1/*3A, and 1 as *1/*3C. Accordingly, correlation analyses between genotype and azathioprine metabolites were performed and reported only for *TPMT* rs1142345, which was common between *3A and *3C alleles.

The *TPMT* rs1142345 variant (76 wild type, 10 heterozygous) was associated with increased concentrations of both DNA-TG and TGN (Kruskal–Wallis *p*-value = 0.024 and *p*-value = 0.00038, Figure 6a,b, respectively); however, for *PACSIN2* rs2413739 (37 wild types, 34 heterozygous, 16 homozygous variants), no significant associations with azathioprine-active metabolites were found. Moreover, the disease activity score was differently distributed on the basis of *PACSIN2* rs2413739 genotype (logistic regression not adjusted for repeated observations *p*-value = 0.04, Figure S9); a similar trend was found after adjusting for repeated measures in each patient (linear mixed effect model *p*-value = 0.049).

**Figure 6:**
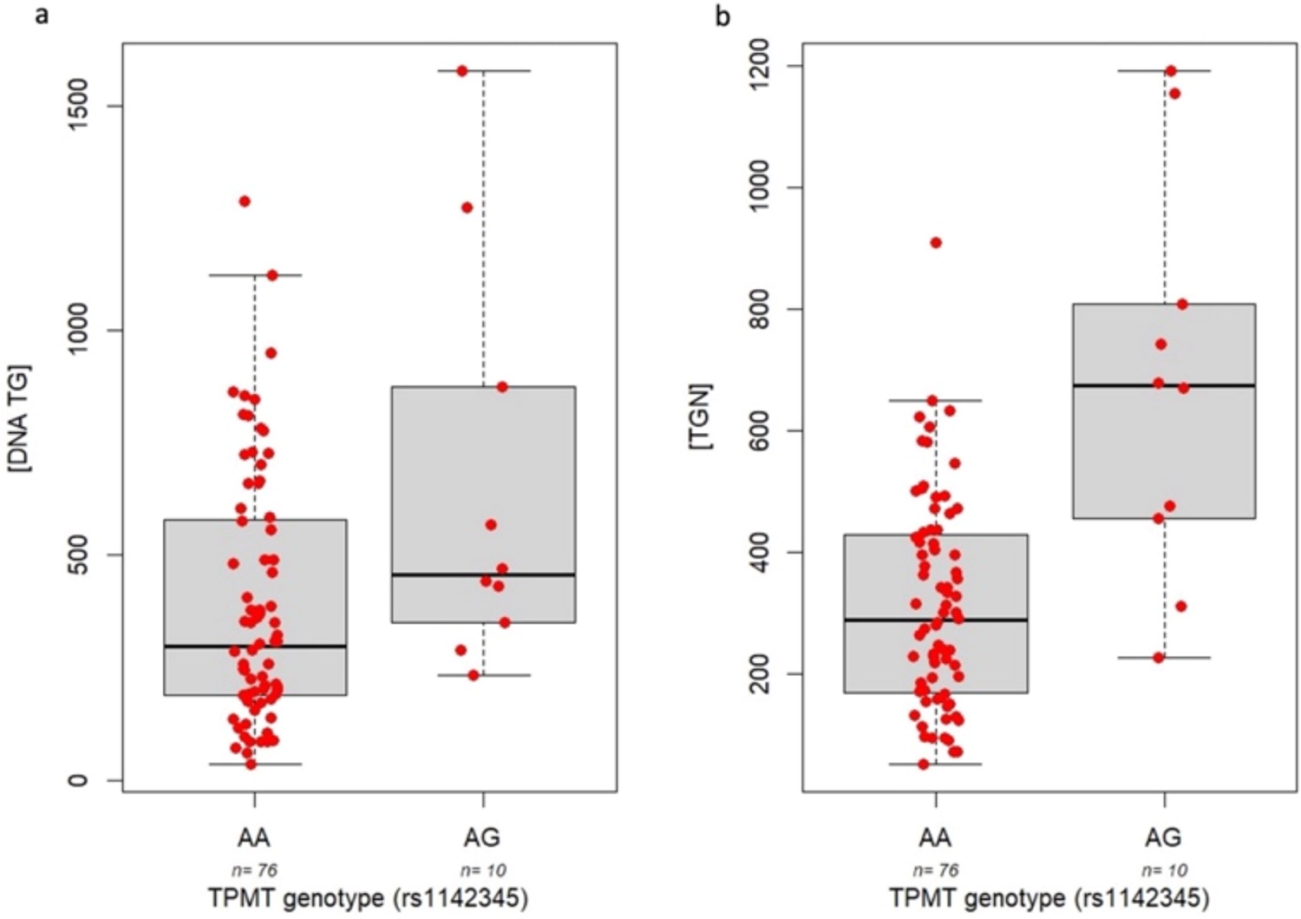
Effect of *TPMT* genetic variant rs1142345 (76 wild type, 10 heterozygous) on the levels of DNA-TG (AA median = 296.1, IQR = 391.9; AG median = 457.24, IQR = 523.7), Kruskal–Wallis *p*-value = 0.034 (panel a); and effect of *TPMT* genetic variant rs1142345 (80 wild type, 6 heterozygous) on RBC TGN levels (AA median = 287.3, IQR = 259.5; AG median = 674, IQR = 353), Kruskal–Wallis *p*-value = 0.034 (panel b).

## Discussion

This study focused on VEO-IBD pediatric patients, who represent a particular IBD subgroup with clinical and genetic peculiarities compared to IBD pediatric patients with a later onset.^2,11^ The reported results highlighted the key role of age in azathioprine metabolism, confirming that VEO-IBD patients require higher azathioprine doses than older patients. Moreover, the potential role of azathioprine metabolites DNA-TG and TGN as drug biomarkers has emerged. In particular, no significant associations between patients’ gender or IBD type on the concentration of both DNA-TG and TGN were detected, whereas an important impact of age was found, confirming its important effect on both azathioprine dose and pharmacokinetics. Despite the standard azathioprine dose for IBD pediatric patients being 2–2.5 mg/kg, it was found that this dosage is not appropriate for VEO-IBD subjects, who require higher azathioprine to achieve disease remission.^12^ Epigenetic factors, such as DNA methylation, change with growth and may affect drug biotransformation.^13^ Recently, we demonstrated that VEO-IBD patients showed a lower methylation of the cg22736354 CpG site, located on the *TPMT* promoter downstream neighboring region, associated with reduced azathioprine inactivation and increased TGN concentrations.^14^ Accordingly, the reported evidence confirmed that VEO-IBD subjects required higher azathioprine doses compared to older IBD pediatric patients and presented increased levels of both TGN and DNA-TG azathioprine metabolites, particularly after adjustment for the administered drug dosage, indicating a possible increased azathioprine metabolism in younger patients, which could be due to a lower *TPMT* methylation and consequent increased TPMT expression.^15^ Interestingly, an age scalar effect for RBC TGN/azathioprine dose ratio and the DNA-TG/azathioprine dose ratio in the analyzed cohort was detected, which could be due to the proportional contribution of age-dependent epigenetic factors involved in drug biotransformation mechanisms.^16^

The recent development of methods for measuring incorporated DNA-TG by LC-MS/MS may provide a more precise, faster, and simpler indication of mercaptopurine efficacy compared to RBC TGN concentration.^9,10^ The measurement of DNA-TG levels requires lower blood volumes of patients, and shows similar cost as that of RBC TGN detection, encouraging its possible use as an azathioprine efficacy biomarker.^17^ From a pharmacokinetics point of view, in this study, a positive association between the amount of incorporated DNA-TG and RBC TGN levels was found, suggesting that both TGN and DNA-TG could be considered good markers of azathioprine metabolism in this cohort. This is the first study to evaluate an association between DNA-TG and disease activity in pediatric IBD. Patients with a higher disease activity presented increased levels of DNA-TG, indicating that their WBC may be more resistant to azathioprine cytotoxic effects. Although the DNA-TG accumulation was previously associated with reduced proliferation and increased apoptosis in *ex vivo* peripheral CD4^+^ T lymphocyte cultures,^18^ in this study, it was found that higher DNA-TG levels were associated with a higher disease score. This result is noteworthy, especially in light of previous studies, which demonstrated that the primary mechanism of action for thiopurines in IBD involves Rac-1 inhibition rather than DNA-TG incorporation.^19,20^ The elevated DNA-TG levels may indicate that less TGN is available for Rac-1 inhibition, which could explain the observed correlation between higher DNA-TG levels and increased disease activity scores. No associations between RBC TGN and the disease activity score were found in the present cohort, in contrast to what had been observed in another study.^21^ This could be related to the fact that in the present cohort, clinicians previously adjusted the administered azathioprine dose to IBD patients according to their RBC TGN amount.^22,23^ Indeed, no correlations were found between azathioprine dosage and the concentration of its metabolites and between RBC TGN and disease activity. A clear association between azathioprine metabolites and drug dose has not been observed^21^ and this could be likely due to the complex activation of azathioprine and the very long half-life of the metabolites.^22^ The correlation analyses between the azathioprine metabolites and the clinical parameters used to determine azathioprine toxicity revealed that, as expected, both WBC DNA-TG and RBC TGN were negatively correlated with the lymphocyte count and positively correlated with the MCV.^24^ However, only TGN showed a significant negative correlation with neutrophil count, WBC count, and platelet count, demonstrating that in the current study, TGN could be more sensitive to detect azathioprine toxicity than DNA-TG in this pediatric patient cohort. Conversely, previous studies investigating the sensitivity of DNA-TG and TGN for predicting thiopurine-related toxicity demonstrated that DNA-TG was more sensitive in detecting leukopenia than TGN.^25–27^ It is important to note that the cohort analyzed by Yang et al. was composed only of IBD adults,^27^ whereas less than 10% of subjects enrolled by Zhu et al. were under 19 years of age.^25^ Moreover, the current study was not designed to assess the sensitivity of DNA-TG and 6-TGN for predicting thiopurine-related toxicity; therefore, further studies are needed to deeply clarify this issue.

From a pharmacogenetic point of view, our data confirmed an important role of the *TPMT* rs1142345 variant in azathioprine metabolism in subjects with IBD,^28^ leading to increased drug metabolite levels, as previously demonstrated.^21^ Also, an interesting contribution of the *PACSIN2* rs2413739 variant with the disease activity scores was found, according to previous results on a different IBD pediatric cohort, where a higher disease activity score was detected in patients carrying the *PACSIN2* rs2413739 variant.^28^ Consistently, previous results detected in pediatric patients affected by acute lymphoblastic leukemia undergoing thiopurine treatment presented a higher risk of thiopurine-gastrointestinal toxicity development in the presence of the *PACSIN2* rs2413739 polymorphism.^29^

This study has some limitations to consider, such as the discrepancy in the numerosity of the three groups of IBD patients used for the analyses: the VEO-IBD cohort and the group of children between 6 and 12 years is smaller than the adolescents’ cohort. It is necessary to take into consideration that repeated samples were not available for all patients; indeed, all analyses were also performed adjusting for the repeated measures.

Together, the reported data confirmed the important role of age in azathioprine pharmacokinetics, which should be considered for personalization of pediatric IBD therapy, and demonstrated that both TGN and DNA-TG could be considered good markers of azathioprine metabolism.

## Materials and Methods

### Patients

This study was conducted in accordance with the principles of the Declaration of Helsinki. Patients were enrolled from 2018 to 2021, and a multicentric case-control observational study was performed after the approval of the local Ethics Committee (Protocol number 31342, 16 January 2018); all the enrolled subjects or their guardians signed an informed consent form to join the study. IBD diagnosis was performed according to the Porto criteria, and patients were classified according to the Paris Classification.^30,31^ Subjects younger than 6 years were considered VEO-IBD cases compared to children between 6 and 12 years old and patients between 12 and 18 years old, who were considered adolescents. The exclusion criteria included concomitant therapy with antitumor necrosis factor biological agents, colostomy, fulminant colitis, and the presence of any of the following conditions: infections (e.g., HIV), tumors, organ transplant, kidney, liver, hematological, endocrine, cardiac, neurological, or cerebral diseases. Clinical disease activity was assessed using pediatric Crohn’s disease activity index (PCDAI) and pediatric ulcerative colitis activity index (PUCAI) for CD and UC patients, respectively.^14^

Blood samples for azathioprine metabolite measurement and genotyping were taken at the appropriate clinic visit. The timing of metabolite level measurement was determined by the clinical setting of azathioprine administration at the hospital: generally, azathioprine metabolite levels were measured after 3, 6, and 12 months of treatment and then every year. Patients were treated with a dose-escalating strategy to reduce the risk of adverse events, starting from a relatively high dose (median of 2 mg/kg). At subsequent follow-up visits (every 3 months), the dose was increased or reduced to obtain the optimal clinical response; the criteria used to increase or reduce the dose of azathioprine were the level of disease activity and laboratory parameters used to monitor azathioprine toxicity (in particular, leukocytes, erythrocytes, and platelet counts; hemoglobin concentration; mean corpuscular volume; liver enzymes alanine aminotransferase, aspartate aminotransferase, and γ-glutamyltransferase; and amylase levels). Moreover, according to current guidelines, TPMT genotypes and RBC TGN concentration were shared with clinicians in order to allow increased monitoring of efficacy and adverse events.

In order to have azathioprine-active nucleotides at the steady state concentration, blood samples of patients treated with azathioprine for at least 90 days were considered. A concentration of TGN <50 pmol/8 × 10^8^ erythrocytes was considered a signal of inadequate treatment compliance, leading to sample exclusion.

### DNA Extraction

The peripheral blood samples were processed for genomic DNA extraction using a commercial kit (GenElute Blood Genomic DNA Kit, Merck) according to the manufacturer’s instructions.

### Measurement of Azathioprine Metabolites

Azathioprine TGN metabolites were measured in patient erythrocytes using the HPLC assay on an Agilent Technologies 1260 HPLC instrument, as previously described^34 (PMID: 36557210)^. Metabolite levels were expressed as pmol/8 × 10^8^ red blood cells (RBC). Levels of DNA-TG were quantified after enzyme digestion of genomic DNA by using an LC-MS/MS assay readapted from a previously published method.^32,33^

### Genotyping

DNA samples were genotyped using the TaqMan SNP genotyping system for *TPMT* rs1142345 (C_19567_20, Applied Biosystem), *TPMT* rs1800460 (C_30634116_20, Applied Biosystem), *TPMT* rs1800462 (C_12091552_30, Applied Biosystem), and *PACSIN2* rs2413739 (C_2503304_20, Applied Biosystem).

### Statistical Analyses

For all analyses, normality of the variables was tested using the Shapiro test. Statistical analyses were performed by Kruskal–Wallis’ test, Spearman’s tests, linear mixed effect model analysis, and logistic regression test using R software version 4.3.1

## Supplementary Material



## Notes

Normality analyses for the continuous variables (Figure S1); effect of IBD type on DNA-TG and TGN concentrations (Figure S2); effect of patient’s gender on DNA-TG and TGN concentrations (Figure S3); associations between azathioprine dose and the concentration of WBC DNA-TG or RBC TGN (Figure S4); association between the disease activity score and DNATG/azathioprine dose ratio (Figure 5); association between azathioprine dose and the disease activity score (Figure S6); correlation analyses between DNA-TG levels and lymphocytes count, amylase, and mean corpuscular volume (Figure S7); correlation analyses between TGN levels and WBC count, neutrophils count, lymphocytes count, platelet count, and MCV (Figure S8); effect of the PACSIN2 genetic variant rs2413739 on the levels of IBD disease activity scores (Figure S9); patients’ genotypes for TPMT rs1142345, rs1800460 (Table S1) (PDF)

## Abbreviations

CD: Crohn’s disease
IBD: inflammatory bowel disease
IQR: interquartile range
PUCAI: pediatric ulcerative colitis activity index
PCDAI: pediatric Crohn’s disease activity index
UC: ulcerative colitis
VEO-IBD: very early onset IBD

## References

[1] Yu Y. R., Rodriguez J. R. (2017). Clinical
presentation of Crohn’s, ulcerative
colitis, and indeterminate colitis: Symptoms, extraintestinal manifestations,
and disease phenotypes. Semin. Pediatr. Surg..

[2] Holbein C. E., Plevinsky J., Patel T., Conrad M. C., Kelsen J. R. (2021). Pediatric
Global Health in Children with Very Early-Onset Inflammatory Bowel
Disease. J. Pediatr. Psychol..

[3] Ouahed J., Spencer E., Kotlarz D., Shouval D. S., Kowalik M., Peng K., Field M., Grushkin-Lerner L., Pai S. Y., Bousvaros A., Cho J., Argmann C., Schadt E., Mcgovern D. P. B., Mokry M., Nieuwenhuis E., Clevers H., Powrie F., Uhlig H., Klein C., Muise A., Dubinsky M., Snapper S. B. (2020). Very Early
Onset
Inflammatory Bowel Disease: A Clinical Approach With a Focus on the
Role of Genetics and Underlying Immune Deficiencies. Inflammatory Bowel Dis..

[4] Ruemmele F. M., Turner D. (2014). Differences in the
management of pediatric and adult
onset ulcerative colitis--lessons from the joint ECCO and ESPGHAN
consensus guidelines for the management of pediatric ulcerative colitis. J. Crohn’s Colitis.

[5] Adam L., Phulukdaree A., Soma P. (2018). Effective long-term solution to therapeutic
remission in Inflammatory Bowel Disease: Role of Azathioprine. Biomed. Pharmacother..

[6] Franca R., Stocco G., Favretto D., Giurici N., Del Rizzo I., Locatelli F., Vinti L., Biondi A., Colombini A., Fagioli F., Barisone E., Pelin M., Martellossi S., Ventura A., Decorti G., Rabusin M. (2020). PACSIN2 rs2413739
influence
on thiopurine pharmacokinetics: validation studies in pediatric patients. Pharmacogenomics J..

[7] Stocco G., Martelossi S., Arrigo S., Barabino A., Aloi M., Martinelli M., Miele E., Knafelz D., Romano C., Naviglio S., Favretto D., Cuzzoni E., Franca R., Decorti G., Ventura A. (2017). Multicentric Case-Control Study on
Azathioprine Dose and Pharmacokinetics in Early-onset Pediatric Inflammatory
Bowel Disease. Inflammatory Bowel Dis..

[8] Lennard L., Singleton H. J. (1992). High-performance liquid chromatographic assay of the
methyl and nucleotide metabolites of 6-mercaptopurine: quantitation
of red blood cell 6-thioguanine nucleotide, 6-thioinosinic acid and
6-methylmercaptopurine metabolites in a single sample. J. Chromatogr. B:Biomed. Sci. Appl..

[9] Choi R., Chun M. R., Park J., Lee J. W., Ju H. Y., Cho H. W., Hyun J. K., Koo H. H., Yi E. S., Lee S. Y. (2021). Quantification of
Thioguanine in DNA Using Liquid Chromatography-Tandem
Mass Spectrometry for Routine Thiopurine Drug Monitoring in Patients
With Pediatric Acute Lymphoblastic Leukemia. Ann. Lab. Med..

[10] Coulthard S. A., Berry P., McGarrity S., Ansari A., Redfern C. P. F. (2016). Liquid
chromatography-mass spectrometry for measuring deoxythioguanosine
in DNA from thiopurine-treated patients. J.
Chromatogr. B.

[11] Levine A. E., Mark D., Smith L., Zheng H. B., Suskind D. L. (2023). Pharmacologic
Management of Monogenic and Very Early Onset Inflammatory Bowel Diseases. Pharmaceutics.

[12] Grossman A. B., Noble A. J., Mamula P., Baldassano R. N. (2008). Increased
dosing requirements for 6-mercaptopurine and azathioprine in inflammatory
bowel disease patients six years and younger. Inflammatory Bowel Dis..

[13] Cascorbi I., Schwab M. (2016). Epigenetics in Drug
Response. Clin. Pharmacol. Ther..

[14] Selvestrel D., Stocco G., Aloi M., Arrigo S., Cardile S., Cecchin E., Congia M., Curci D., Gatti S., Graziano F., Langefeld C. D., Lucafò M., Martellossi S., Martinelli M., Pagarin S., Scarallo L., Stacul E. F., Strisciuglio C., Thompson S., Zuin G., Decorti G., Bramuzzo M. (2023). DNA methylation
of the TPMT gene
and azathioprine pharmacokinetics in children with very early onset
inflammatory bowel disease. Biomed. Pharmacother..

[15] Lucafò M., Franca R., Selvestrel D., Curci D., Pugnetti L., Decorti G., Stocco G. (2018). Pharmacogenetics
of treatments for
inflammatory bowel disease. Expert Opin. Drug
Metab. Toxicol..

[16] Peng L., Zhong X. (2015). Epigenetic regulation
of drug metabolism and transport. Acta Pharm.
Sin B.

[17] Bayoumy A. B., Ansari A. R., Mulder C. J. J., Schmiegelow K., Florin T., De Boer N. K. H. (2024). Innovating Thiopurine Therapeutic
Drug Monitoring: A Systematic Review and Meta-Analysis on DNA-Thioguanine
Nucleotides (DNA-TG) as an Inclusive Biomarker in Thiopurine Therapy. Clin. Pharmacokinet..

[18] Toyonaga T., Kobayashi T., Kuronuma S., Ueno A., Kiyohara H., Okabayashi S., Takeuchi O., Redfern C. P. F., Terai H., Ozaki R., Sagami S., Nakano M., Coulthard S. A., Tanaka Y., Hibi T. (2021). Increased DNA-incorporated thiopurine
metabolite as a possible mechanism for leukocytopenia through cell
apoptosis in inflammatory bowel disease patients with NUDT15 mutation. J. Gastroenterol..

[19] Deben D. S., van Adrichem A. J., Drent R., Puts S., Pelzer K. E. J. M., van Bodegraven A. A., Wong D. R., Leers M. P. G. (2022). Rac1/pSTAT3
expression: A pharmacodynamic marker panel as a first step toward
optimization of thiopurine therapy in inflammatory bowel disease patients. Cytometry, Part A.

[20] Seinen M. L., van Nieuw Amerongen G. P., de Boer N. K., Mulder C. J., van Bezu J., van Bodegraven A. A. (2016). Rac1 as
a Potential Pharmacodynamic
Biomarker for Thiopurine Therapy in Inflammatory Bowel Disease. Ther. Drug Monit..

[21] Lucafò M., Stocco G., Martelossi S., Favretto D., Franca R., Malusà N., Lora A., Bramuzzo M., Naviglio S., Cecchin E., Toffoli G., Ventura A., Decorti G. (2019). Azathioprine
Biotransformation in Young Patients with Inflammatory Bowel Disease:
Contribution of Glutathione-S Transferase M1 and A1 Variants. Genes.

[22] Wright S., Sanders D. S., Lobo A. J., Lennard L. (2004). Clinical significance
of azathioprine active metabolite concentrations in inflammatory bowel
disease. Gut.

[23] Nguyen T. V., Vu D. H., Nguyen T. M., Lachaux A., Boulieu R. (2013). Relationship
between azathioprine dosage and thiopurine metabolites in pediatric
IBD patients: identification of covariables using multilevel analysis. Ther. Drug Monit..

[24] Heerasing N. M., Ng J. F., Dowling D. (2016). Does lymphopenia
or macrocytosis
reflect 6-thioguanine levels in patients with inflammatory bowel disease
treated with azathioprine or 6-mercaptopurine?. Intern. Med. J..

[25] Zhu X., Chao K., Yang T., Wang X. D., Guan S., Tang J., Xie W., Yu A. M., Yang Q. F., Li M., Yang S., Diao N., Hu P., Gao X., Huang M. (2022). DNA-Thioguanine Nucleotides as a Marker for Thiopurine Induced Late
Leukopenia after Dose Optimizing by NUDT15 C415T in Chinese Patients
with IBD. Clin. Pharmacol. Ther..

[26] Bayoumy A.
B., Derijks L. J. J., de Boer N. K. H. (2025). DNA-Thioguanine (DNA-TG) Is a Promising
Novel Method to Predict Adverse Events to Thiopurine in Inflammatory
Bowel Disease. Inflammatory Bowel Dis..

[27] Yang T., Chao K., Zhu X., Wang X. D., Chan S., Guan Y. P., Mao J., Li P., Guan S. X., Xie W., Xiang G., Huang M. (2024). Early proactive monitoring of DNA-thioguanine
in patients with Crohn’s disease predicts thiopurine-induced
late leucopenia in NUDT15/TPMT normal metabolizers. World J. Gastroenterol..

[28] Franca R., Zudeh G., Pagarin S., Rabusin M., Lucafò M., Stocco G., Decorti G. (2019). Pharmacogenetics of
thiopurines. Cancer Drug Resist..

[29] Stocco G., Yang W., Crews K. R., Thierfelder W. E., Decorti G., Londero M., Franca R., Rabusin M., Valsecchi M. G., Pei D., Cheng C., Paugh S. W., Ramsey L. B., Diouf B., McCorkle J. R., Jones T. S., Pui C., Relling M. V., Evans W. E. (2012). PACSIN2
polymorphism influences TPMT
activity and mercaptopurine-related gastrointestinal toxicity. Hum. Mol. Genet..

[30] Levine A., Griffiths A., Markowitz J., Wilson D. C., Turner D., Russell R. K., Fell J., Ruemmele F. M., Walters T., Sherlock M., Dubinsky M., Hyams J. S. (2011). Pediatric modification
of the Montreal classification for inflammatory bowel disease: the
Paris classification. Inflammatory Bowel Dis..

[31] Levine A., Koletzko S., Turner D., Escher J. C., Cucchiara S., de Ridder L., Kolho K. L., Veres G., Russell R. K., Paerregaard A., Buderus S., Greer M. C., Dias J. A., Veereman-Wauters G., Lionetti P., Sladek M., De Carpi J. M., Staiano A., Ruemmele F. M., Wilson D. C. (2014). European Society
of Pediatric Gastroenterology, Hepatology, and Nutrition. ESPGHAN
revised porto criteria for the diagnosis of inflammatory bowel disease
in children and adolescents. J. Pediatr. Gastroenterol.
Nutr..

[32] Nishii R., Moriyama T., Janke L. J., Yang W., Suiter C. C., Lin T. N., Li L., Kihira K., Toyoda H., Hofmann U., Schwab M., Takagi M., Morio T., Manabe A., Kham S., Jiang N., Rabin K. R., Kato M., Koh K., Yeoh A. E., Hori H., Yang J. J. (2018). Preclinical evaluation
of. Blood.

[33] Jacobsen J. H., Schmiegelow K., Nersting J. (2012). Liquid chromatography-tandem mass
spectrometry quantification of 6-thioguanine in DNA using endogenous
guanine as internal standard. J. Chromatogr.
B.

