# Supplementary material for: Azathioprine Metabolites in Erythrocytes and DNA for Therapy Monitoring in Very Early Onset Inflammatory Bowel Disease Pediatric Patients
Source: ACS Pharmacol Transl Sci. 2025 Jun 5;8(7):2009–17. doi: 10.1021/acsptsci.5c00135 (PMC12260932; doi:10.1021/acsptsci.5c00135)
Supplement: Supplementary file 1 [file pt5c00135_si_001.pdf]

SUPPORTING INFORMATION

Azathioprine metabolites in erythrocytes and DNA for therapy monitoring in very early onset inflammatory bowel disease pediatric patients.

Giulia Zudeh[1‡], Martina Franzin[1‡], Marianna Lucafò[2*], Matteo Bramuzzo[3], Debora Curci[1], Jun J. Yang[4], Maud Maillard[4], Giuliana Decorti[5], Gabriele Stocco[1,5]

AUTHOR ADDRESS

[1]Department of Translational and Advanced Diagnostics, Institute for Maternal and Child Health I.R.C.C.S. Burlo Garofolo, Trieste, 34137, Italy

[2]Department of Life Sciences, University of Trieste, 34128, Trieste, Italy

[3]Department of Gastroenterology, Digestive Endoscopy and Nutrition Unit, Institute for Maternal and Child Health I.R.C.C.S. Burlo Garofolo, Trieste, 34137, Italy

[4]Department of Pharmacy and Pharmaceutical Sciences, St. Jude Children's Research Hospital, Memphis, 38105-3678 TN, USA

[5]Department of Medical, Surgical and Health Sciences, University of Trieste, Trieste, 34129, Italy

‡these authors contributed equally to the study

*corresponding author, e-mail address: mlucafo@units.it

TABLE OF CONTENT

Figure S1	Normality analyses for the continuous variables	Page S3
Figure S2	Effect of IBD type on DNA-TG and TGN concentrations	Page S4
Figure S3	Effect of patient's gender on DNA-TG and TGN concentrations	Page S4
Figure S4	Associations between azathioprine dose and the concentration of WBC DNA-TG or RBC TGN	Page S5
Figure S5	Association between disease activity score and DNA-TG/azathioprine dose ration	Page S5
Figure S6	Association between azathioprine dose and the disease activity score	Page S6
Figure S7	Correlation analyses between DNA-TG levels and lymphocytes count, amylase and Mean Corpuscular Volume	Page S7
Figure S8	Correlation analyses between TGN levels and WBC count, neutrophils count,lymphocytes count, platelet count and MCV	Page S8
Figure S9	Effect of PACSIN2 genetic variant rs2413739 on the levels of IBD disease activity score	Page S9
Table S1	Patients' genotypes for *TPMT* rs1142345, rs1800460,	Page S9

	rs1800462, *PACSIN2* rs2413739 and Hardy-Weinberg's test results	

Supplementary Figures

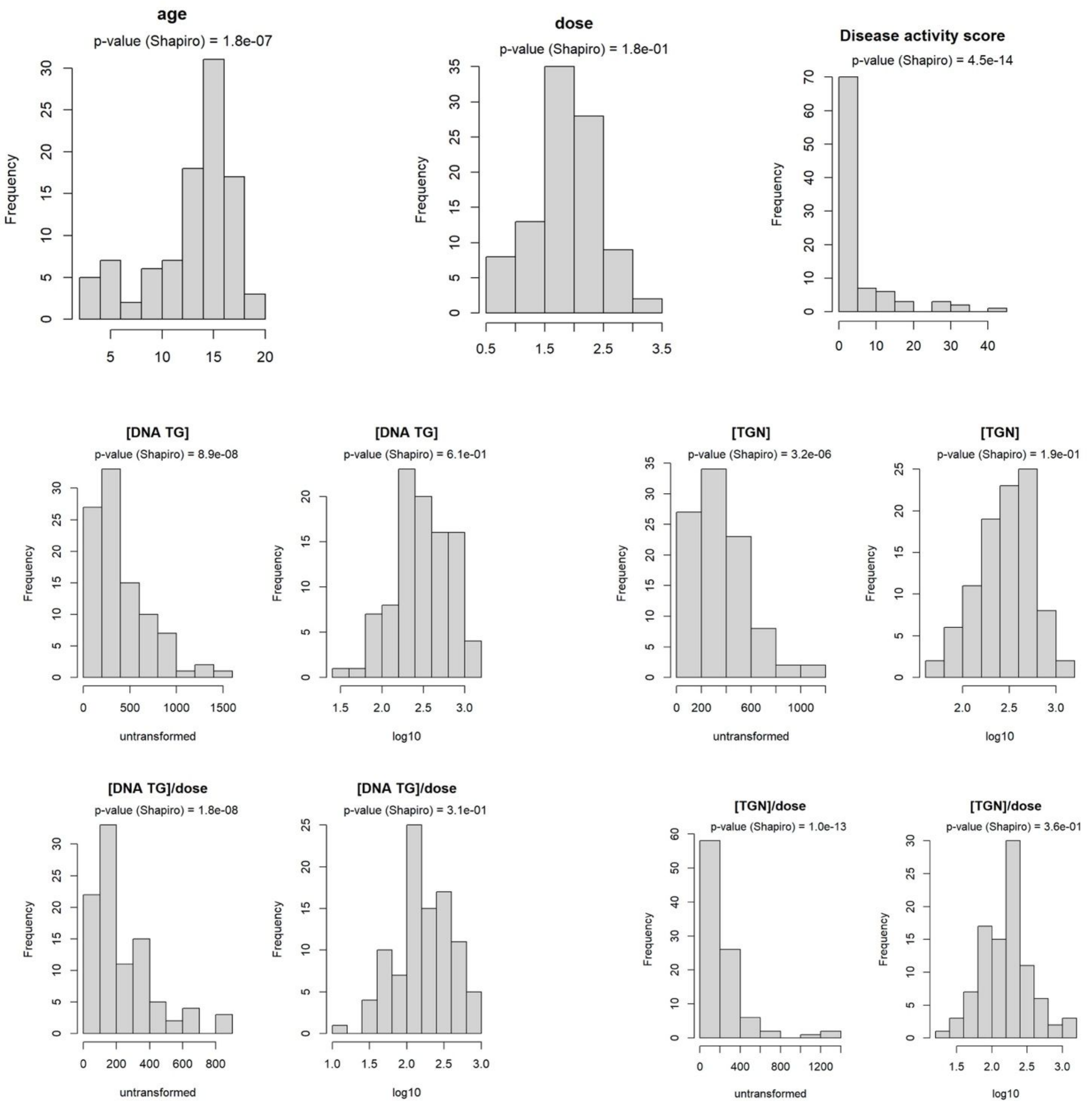

Figure S1. Shapiro test for testing the data normality for the continuous variables.

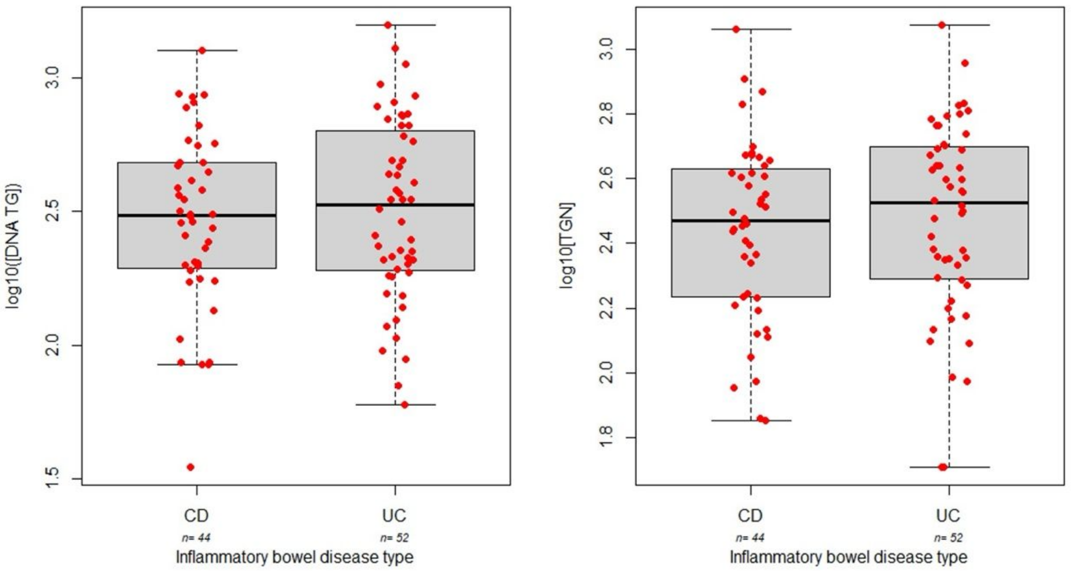

Figure S2. Effect of IBD type on DNA-TG and TGN concentrations (Wilcoxon p = 0.66 and p = 0.4, respectively). CD: Crohn disease, UC: Ulcerative Colitis.

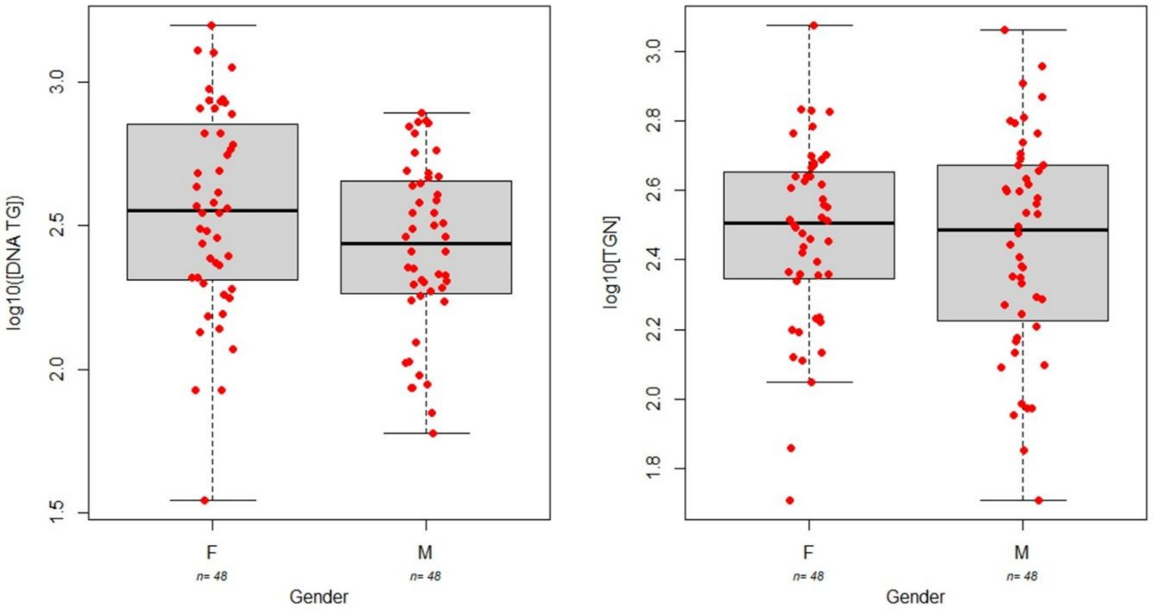

Figure S3. Effect of patient's gender on DNA-TG and TGN concentrations (Wilcoxon p = 0.06 and n p = 0.7, respectively). F: female, M: male

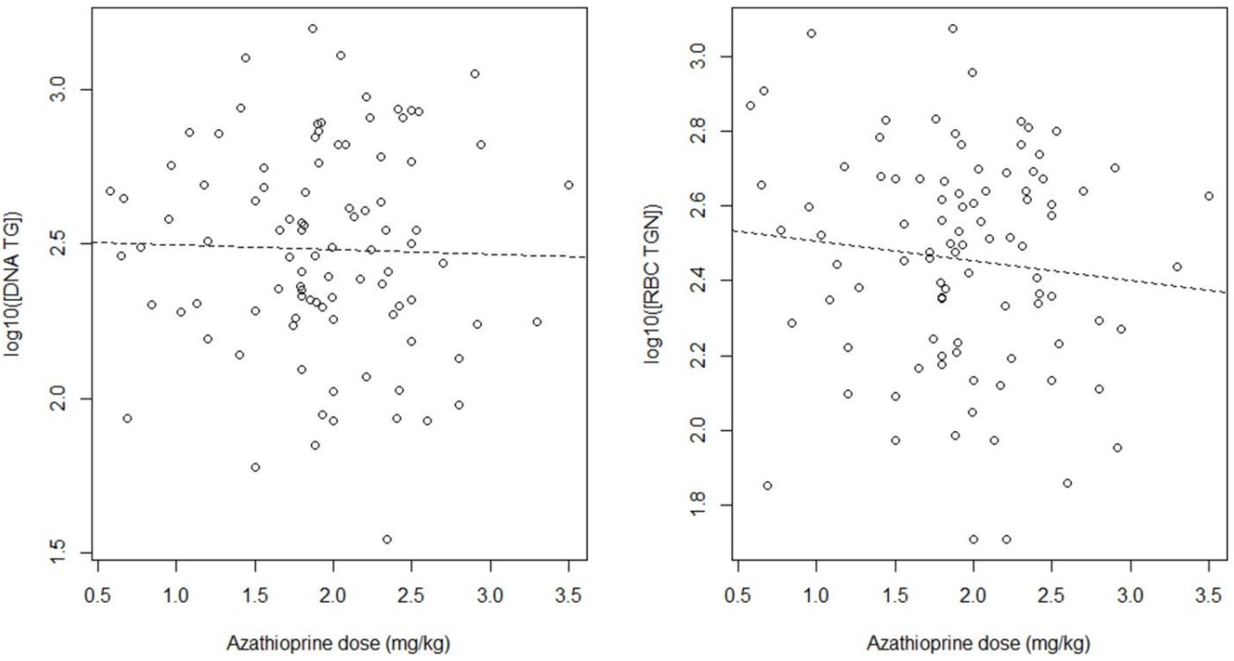

Figure S4. Associations between azathioprine dose and the concentration of WBC DNA-TG, Spearman ρ = -0.024, p = 0.82 (panel a) and RBC TGN concentrations, Spearman ρ = -0.086, p = 0.41 (panel b)

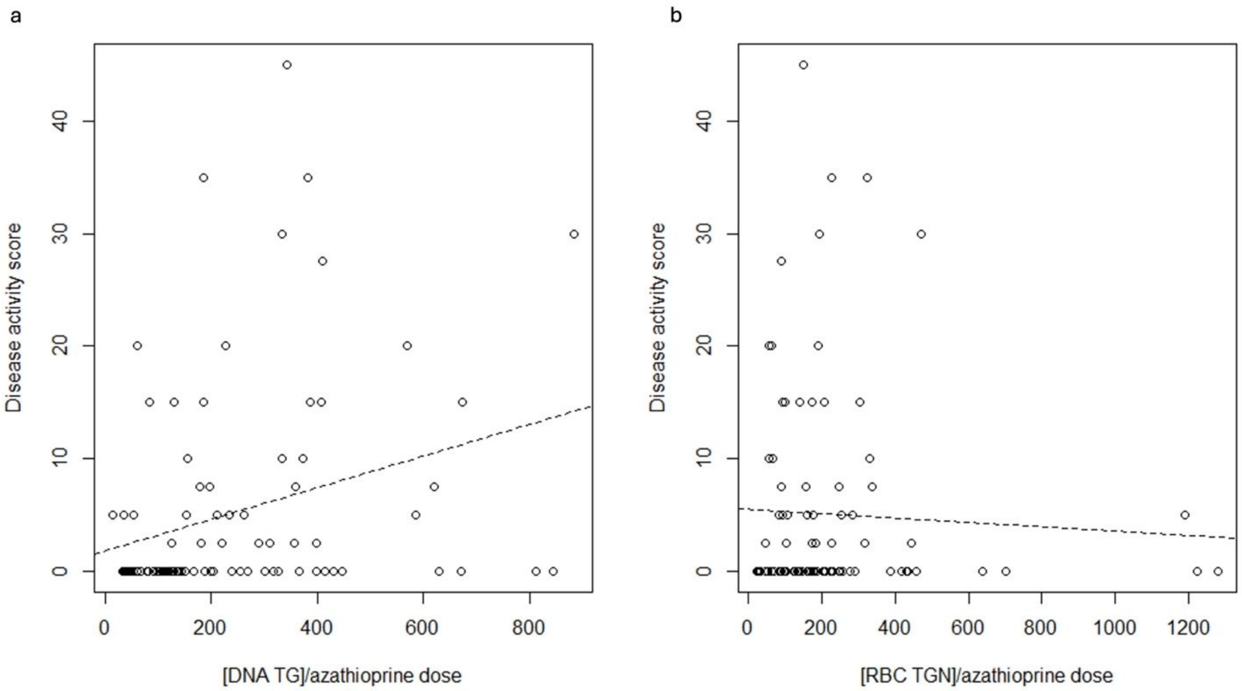

Figure S5. Association between disease activity score and DNA-TG/azathioprine dose ration, Spearman ρ = 0.35, p = 0.00059 (panel a) and TGN/azathioprine dose, Spearman ρ = 0.016, p = 0.88 (panel b).

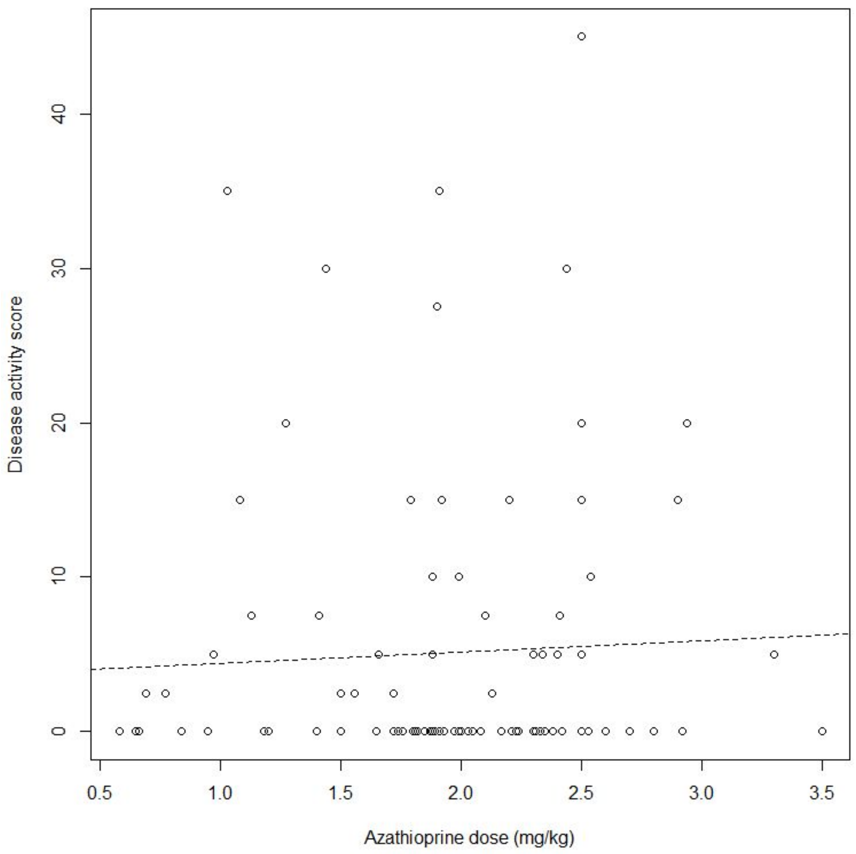

Figure S6. Association between azathioprine dose and the disease activity score, Spearman ρ = 0.031, p = 0.77.

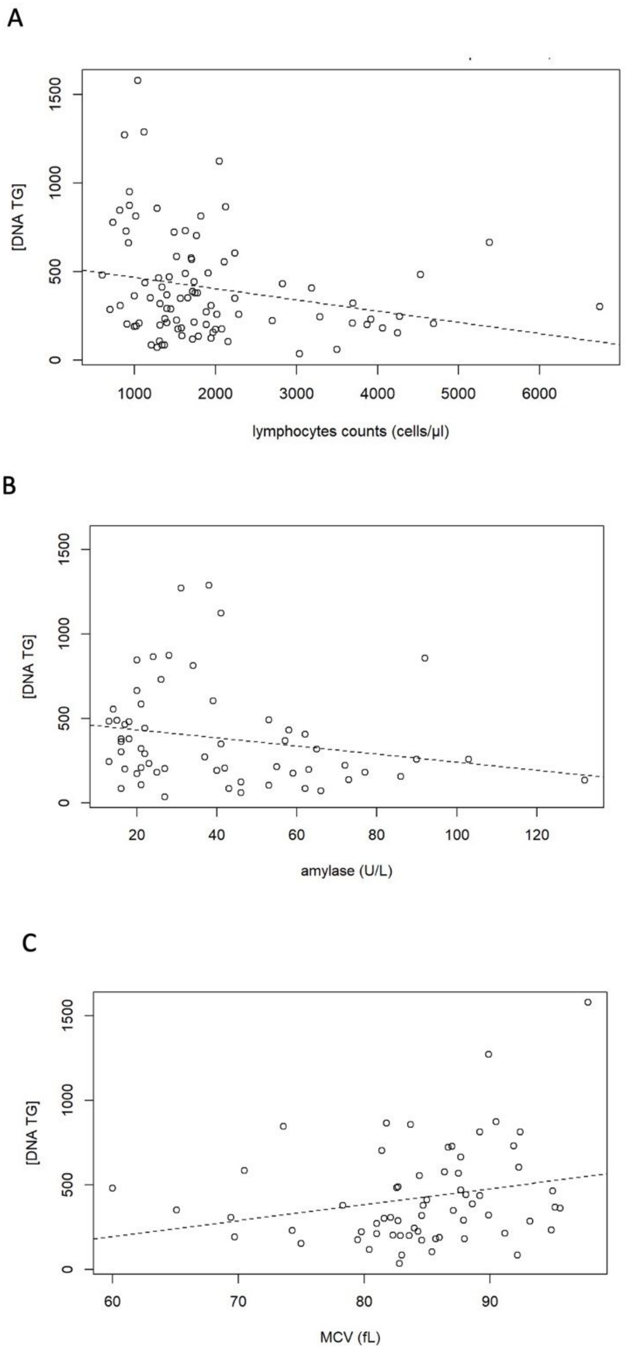

Figure S7. Correlation analyses between DNA-TG levels and lymphocytes count (Spearman ρ = -0.24, p = 0.019, panel a), amylase (Spearman ρ = -0.3, p = 0.026, panel b) and Mean Corpuscular Volume (MCV, Spearman ρ = 0.24, p = 0.05, panel c).

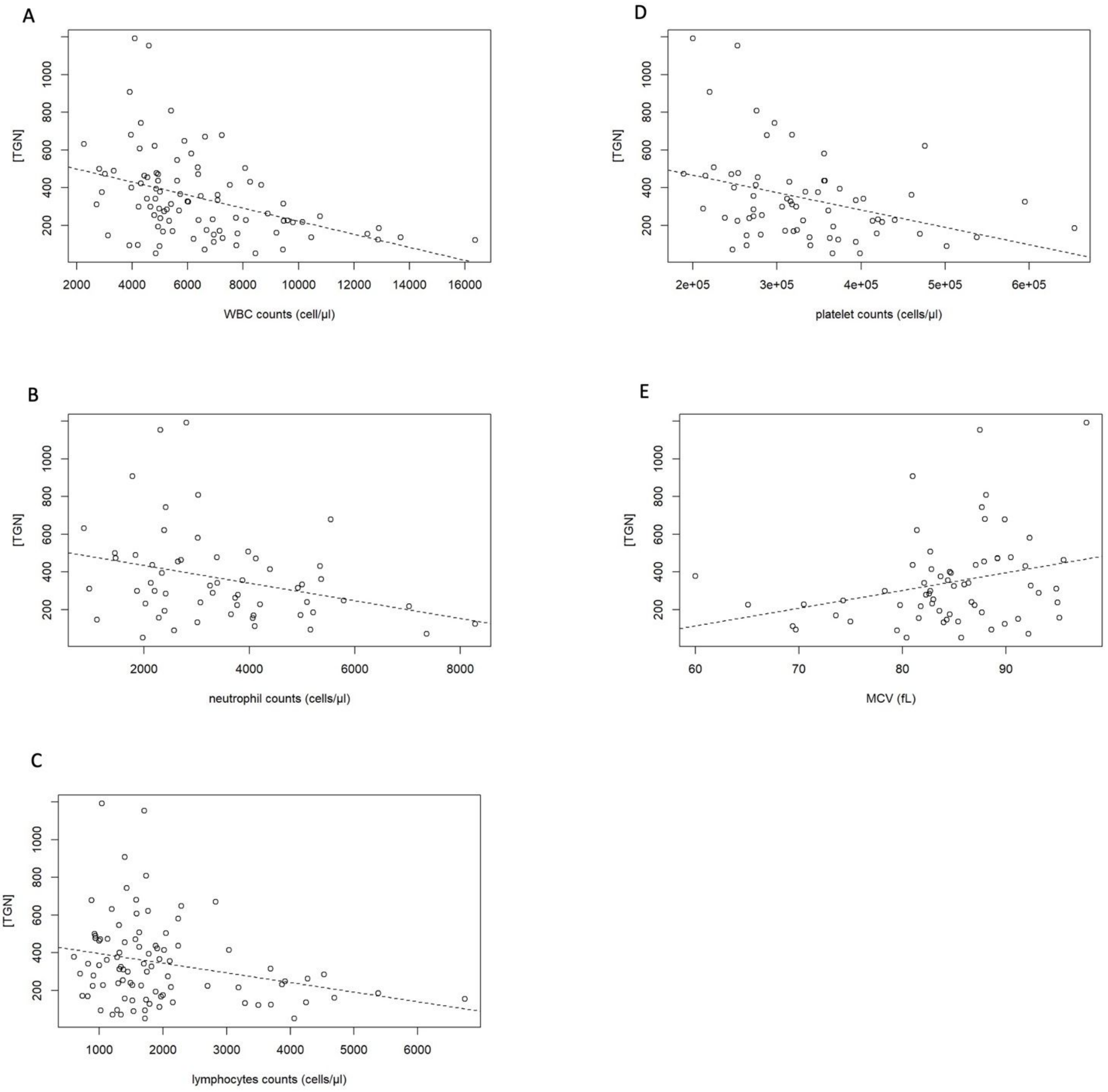

Figure S8. Correlation analyses between TGN levels and WBC count (Spearman ρ = -0.4, p = 1.48 x 10^{-5}, panel a), neutrophils count (Spearman ρ = -0.3, p = 0.03, panel b), lymphocytes count (Spearman ρ = -0.2, p = 0.03, panel c), platelet count (Spearman ρ = -0.4, p = 0.0015, panel d) and MCV (Spearman ρ = 0.3, p = 0.02, panel e).

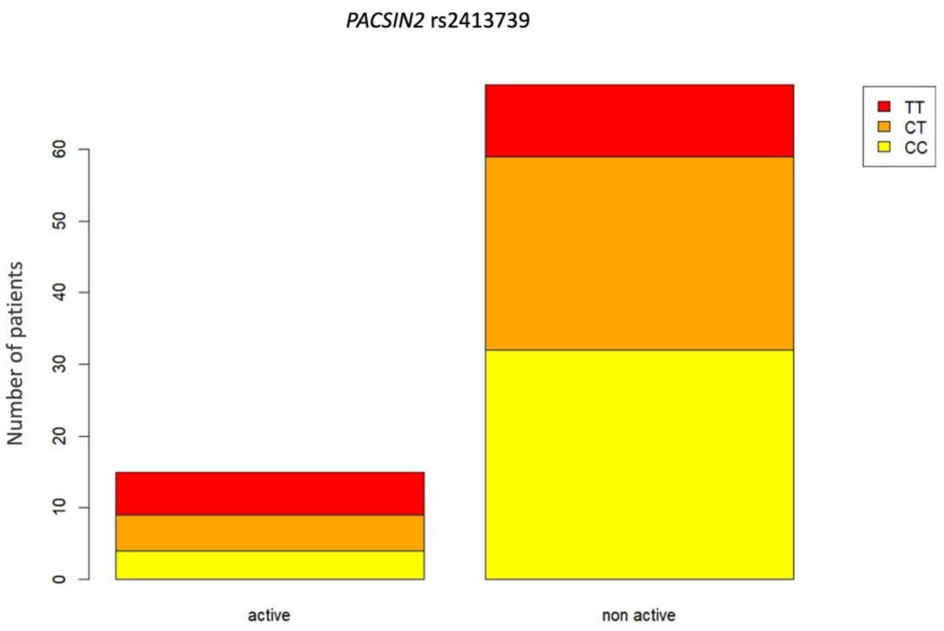

Figure S9. Effect of PACSIN2 genetic variant rs2413739 (37 wild type, 34 heterozygous, 16 homozygous mutated) on the levels of IBD disease activity score, logistic regression not adjusted for repeated observations p-value = 0.040.

Supplementary Table

Genotype	Wild-type	Heterozygous	Homozygous variant	Hardy-Weinberg test
TPMT rs1142345	55	6	-	p = 0.32
TPMT rs1800460	56	5	-	p = 0.18
TPMT rs1800462	61	-	-	p = 1
PACSIN2 rs2413739	24	28	10	p = 0.94

Table S1. Patients' genotypes for *TPMT* rs1142345, rs1800460, rs1800462, *PACSIN2* rs2413739 and Hardy-Weinberg's test results. For *TPMT* alleles patients were classified 55 as *1/*1, 5 as *1/*3A and 1 as *1/*3C.
